# Overwork among resident physicians: national questionnaire survey results

**DOI:** 10.1186/s12909-022-03789-7

**Published:** 2022-10-20

**Authors:** Masatoshi Ishikawa

**Affiliations:** 1 Faculty of Medicine, University of Tsukuba, 1 Chome-1-1 Tennodai, 305-8577 Tsukuba, Ibaraki Japan; 2 Research Institute, Tokyo Healthcare University, Shinagawa, Tokyo, Japan

**Keywords:** Physicians, Burnout, Questionnaire survey, Japan, Work hours

## Abstract

**Background:**

Residents experience the longest working hours among physicians. Thus, it would be beneficial to perform a nationwide survey in Japan on residents’ long work hours and the background factors promoting upper limits on working hours of Japanese residents. The aim of this study was to study or assess the state of physicians’ excessive work hours and its background factors using a questionnaire survey.

**Methods:**

The survey was sent to 924 hospitals. The physicians’ general attributes, work hours and conditions, and employers’ foundational entities were explored. Multiple logistic regression analysis was performed to elucidate the background factors for long work hours.

**Results:**

Of the 4306 resident physicians who responded, 67% had ≥ 60 in-hospital hours/week and 27% had ≥ 80 h/week; 51% were on-call ≥ four times/month. Many of them hoped for increased remuneration. Additionally, female (reference: male, OR: 0.65, 95% CI: 0.55–0.76), 35–40 years old (reference: 25–30 years old, OR: 1.83, 95% CI: 1.32–2.54), childlessness (reference: child, OR: 1.41, 95% CI: 1.12–1.75), surgical specialization (reference: internal medicine, OR: 2.51, 95% CI: 1.96–3.23), neurosurgical specialization (reference: internal medicine, OR: 4.38, 95% CI: 2.92–6.59) and hospitals with 200–400 physicians (reference: <100 physicians, OR: 1.82, 95% CI: 1.12–2.96) exhibited significant correlations with ≥ 80 in-hospital hours/week.

**Conclusion:**

Understanding the factors that increase the likelihood of residents working very long hours could aid in making targeted changes to address the specific concerns. Moreover, reducing working hours to a reasonable limit can improve resident physicians’ health and the quality of care they provide in their community.

## Background

The work hours of Japanese workers are indicated to be longer compared to workers in other countries [1]. The long working hours of physicians, approximately more than 60 h a week, account for 42% of full-time physicians working more than 200 days per year, compared to a 14% average for all other professions [2]. Among physicians, residents (physicians undergoing training in order to become specialists) have the longest working hours [3].

Depression-related suicides and deaths due to ischemic heart disease and cerebrovascular disease caused by overwork are called “karoshi” (death due to overwork) and have become a public health issue specific to East Asia, such as China [4]. Moreover, there is ample evidence regarding the adverse effects of long work hours on health, both in Japan and overseas. According to a systematic review by Bannai et al. [5], long working hours are correlated with depression, anxiety, sleep, and coronary artery disease.

In 2003, the US Accreditation Council for Graduate Medical Education (ACGME) limited residents’ work hours to less than 80 h per week due to its adverse effects on their health and the increased risk of medical error [6, 7]. A systematic review that evaluated this measure concluded that the work-hour restrictions of the ACGME are correlated with an improvement in emotional malaise and burnout syndrome symptoms [8].

Moreover, in Japan, several studies performed at single facilities have indicated the depressive tendencies and burnout of interns and residents [9, 10]. However, unlike those in the US, there have been no restrictions on working hours. Additionally, some physicians not being paid [11] and long working hours [3] have been indicated as issues in the working environment of Japanese residents. However, no nationwide survey studies have been conducted in Japan on resident physicians.

In 2016, the Ministry of Health, Labour and Welfare instituted the “Study Meeting on Physician Labor Reform,” which has been examining measures to promote labor reforms for physicians, and it has disclosed a policy wherein from April 2024, the upper limit on annual overtime work hours for residents will be 1860 h (at par with the US ACGME) [12].

To administratively promote the setting of upper limits on working hours for Japanese residents, it will be useful to perform a nationwide survey on the condition of long work hours for residents and its background factors, and examine topics relevant to the promotion of physician labor reforms.

Therefore, a nationwide questionnaire survey was performed on Japanese residents who work particularly long hours, and it discussed the overworked condition of the resident physicians and background factors associated with its effects to suggest policies for the promotion of labor reforms.

## Methods

### Survey participants

Accounting for the 19 basic areas stipulated by the Japanese Medical Specialty Board, the survey was sent to 924 core hospitals nationwide that had received training program approval for each of the basic areas. Effective responses were received from 4306 residents at 416 hospitals (response rate = 49%). The response rate is related to the % of hospitals from which the responses were received. The number of residents and the location and specialty are unknown. The survey was conducted for 14 days, from 10 October to 23 October 2020.

### Investigation items

A web survey was conducted using a questionnaire. First, the respondents’ attributes (sex, age, basic area of specialization, marital/parental status, income (1 dollar = 125 yen), and foundational entity of employer/no. of beds/no. of full-time doctors/regional classifications) were described. There were four types of foundational entities of the employers: public (except for national universities); national universities; private universities; and private (except for private universities). The regional classifications were divided into Group 1 (urban areas), Group 2 (intermediate areas), and Group 3 (rural areas) based on the combination of the population size and density as of 2019 regarding the 344 secondary care zones nationwide [13].

Next, with regard to the employment status of the residents, the number of hours spent in the hospital, the number of times on duty, etc. were described.

### Statistical analysis

To elucidate the background factors for residents’ long work hours, multiple logistic regression analyses was performed with ≥ 80 in-hospital hours per week as the explanatory variable and sex, age, basic field of specialization, marital/parental status, income, and foundational entity of employer/no. of beds/no. of full-time doctors/regional classifications as response variables. In the statistical analysis, a p value of < 0.05 was considered statistically significant. STATA 15.1 was used in all analyses.

### Ethical considerations

This study was conducted after receiving approval from the medical ethics board of the University of Tsukuba Faculty of Medicine (no. 1498). All methods were performed in accordance with the relevant guidelines and regulations of the Declaration of Helsinki. On the first page of the questionnaire, the purpose of the study and measures for safe data management were explained. Additionally, participants in this study were informed that their participation was entirely voluntary. Data were collected from participants who provided informed consent to participate. The results of the survey were analyzed separately in order to ensure the anonymization and confidentiality of personal information.

## Results

Regarding the respondents’ attributes shown in Tables 1 and 37% were female, 60% were between 30 − 39 years of age, 44% were married, and 21% had children. Additionally, 23% specialized in general internal medicine. The average annual income ranged from ≥ 4 to < 6 million yen. Regarding employer attributes, 54% and 38% worked in large urban areas and national university hospitals, respectively. The number of beds was between 600 and 800, and there were ≥ 600 physicians in most hospitals. Moreover, 70% of the physicians had a part-time job.

**Table 1:** Respondent attributes and in-hospital hours

Attributes of Respondents	n	%		Annual Income (not including part-time work; dollars)n (%)		
Sex				< 16,000	138	3.2
Male	2710	62.9		≥ 16,000–<32,000	1014	23.5
Female	1596	37.1		≥ 32,000–<48,000	1083	25.2
Age (years)				≥ 48,000–<64,000	879	20.4
≥ 25–<30	2587	60.1		≥ 64,000–<80,000	673	15.6
≥ 30–<35	1270	29.5		≥ 80,000–<96,000	343	8.0
≥ 35–<40	220	5.1		≥ 96,000	176	4.1
≥ 40	229	5.3		In-hospital (hours per week)		
Marital Status				< 40 h	78	1.8
Married	1904	44.2		40–60 h	1305	30.3
Not married	2402	55.8		60–80 h	1783	41.4
Parental Status				80–100 h	783	18.2
Children	909	21.1		≥ 100 h	357	8.3
No children	3397	78.9		Foundational Entity of Employer		
Basic Area of Specialization				Public	1224	28.4
General Internal Medicine	979	22.7		National University	1618	37.6
Surgery	437	10.1		Private University	945	21.9
Orthopedic Surgery	329	7.6		Private	519	12.1
Pediatrics	297	6.9		Employer’s Total No. of Beds		
OB/GYN	274	6.4		< 200	63	1.5
Anesthesiology	272	6.3		≥ 200–<400	320	7.4
Otolaryngology	227	5.3		≥ 400–<600	677	15.7
Psychiatry	212	4.9		≥ 600–<800	1412	32.8
Dermatology	187	4.3		≥ 800–<1,000	1072	24.9
Ophthalmology	152	3.5		≥ 1,000	762	17.7
Emergency Medicine	146	3.4		Employer’s No. of Full-time Physicians		
Urology	145	3.4		< 100	421	9.8
Radiology	139	3.2		≥ 100–<200	768	17.8
Neurosurgery	125	2.9		≥ 200–<300	592	13.7
Pathology	118	2.7		≥ 300–<400	733	17.0
Plastic Surgery	105	2.4		≥ 400–<500	476	11.1
General Practice	89	2.1		≥ 600	1,316	30.6
Rehabilitation Medicine	59	1.4		Employer’s Regional Classification		
Clinical Testing	14	0.3		Urban	2315	53.8
Part-time Work				Intermediate	1914	44.4
Yes	2991	69.5		Rural	77	1.8
No	1315	30.5				

As for the weekly in-hospital time, 32%, 41%, and 27% of physicians spent < 60, 60–80 h, and greater than 80 h in the hospital per week, respectively.

Table 2 shows the results of multiple logistic regression analysis with in-hospital time of ≥ 80 h per week as the explanatory variable in order to elucidate the background factors for long work hours of residents.

**Table 2:** Multiple logistic regression analysis with residents’ long work hours as the explanatory variable

	Odds Ratio	95% Confidence Interval	P Value
Sex			
Male	Reference		
Female	0.65	0.55–0.76	< 0.01
Age (years)			
≥ 25–<30	Reference		
≥ 30–<35	1.24	1.05–1.46	0.01
≥ 35–<40	1.83	1.32–2.54	< 0.01
≥ 40	0.77	0.51–1.15	0.20
Marital Status			
Married	Reference		
Not married	1.11	0.93–1.32	0.23
Parental Status			
Children	Reference		
No children	1.41	1.12–1.75	< 0.01
Basic Area of Specialization			
General Internal Medicine	Reference		
Surgery	2.51	1.96–3.23	< 0.01
Orthopedic Surgery	1.29	0.97–1.74	0.08
Pediatrics	1.32	0.98–1.78	0.07
OB/GYN	1.36	0.99–1.87	0.06
Anesthesiology	0.46	0.31–0.69	< 0.01
Otolaryngology	0.69	0.47–1.00	0.05
Psychiatry	0.57	0.38–0.85	< 0.01
Dermatology	0.69	0.45–1.05	0.08
Ophthalmology	0.61	0.39–0.96	0.03
Emergency Medicine	1.11	0.74–1.66	0.62
Urology	1.37	0.93–2.03	0.11
Radiology	0.28	0.15–0.51	< 0.01
Neurosurgery	4.38	2.92–6.59	< 0.01
Pathology	0.46	0.26–0.81	< 0.01
Plastic Surgery	1.22	0.77–1.94	0.40
General Practice	0.81	0.45–1.47	0.49
Rehabilitation Medicine	0.50	0.23–1.08	0.08
Clinical Testing	0.24	0.03–1.95	0.18
Annual Income (dollars)			
< 16,000	Reference		
≥ 16,000–<32,000	0.84	0.54–1.30	0.43
≥ 32,000–<48,000	0.97	0.62–1.52	0.90
≥ 48,000–<64,000	0.79	0.50–1.25	0.31
≥ 64,000–<80,000	0.96	0.60–1.53	0.86
≥ 80,000–<96,000	0.71	0.42–1.17	0.18
≥ 96,000	0.76	0.42–1.36	0.36
Foundational Entity of Employer			
Public	Reference		
National University	0.85	0.62–1.16	0.30
Private University	0.96	0.66–1.40	0.84
Private	1.00	0.76–1.32	0.99
Employer’s Total No. of Beds			
< 200	Reference		
≥ 200–<400	1.45	0.65–3.21	0.36
≥ 400–<600	1.33	0.58–3.04	0.50
≥ 600–<800	1.40	0.59–3.31	0.45
≥ 800–<1,000	1.37	0.57–3.32	0.48
≥ 1,000	1.43	0.59–3.51	0.43
Employer’s No. of Full-time Physicians			
< 100	Reference		
≥ 100–<200	1.25	0.86–1.82	0.25
≥ 200–<300	1.64	1.05–2.55	0.03
≥ 300–<400	1.82	1.12–2.96	0.02
≥ 400–<500	1.56	0.93–2.62	0.10
≥ 600	1.41	0.85–2.34	0.18
Employer’s Regional Classification			
Urban	Reference		
Intermediate	1.00	0.85–1.18	0.98
Rural	0.82	0.41–1.62	0.56

Sex, age (≥ 30–<40 years), no children, surgical/neurosurgical specialization, and number of physicians (≥ 200–<400) exhibited statistically significant correlation as background factors for physicians being in-hospital for ≥ 80 h per week. However, there were no statistically significant correlations with marital status, income, foundational entity of employer, number of beds, and regional characteristics.

## Discussion

First, 68% and 27% of the responding physicians exceeded 60 and 80 h of in-hospital time per week, respectively, elucidating the actual state of overwork for Japanese resident physicians. An in-hospital time of 60 h per week converts to four hours of overtime per day for an 8-hours/day 40-hour week. This is the legal work hour stipulated by the Labor Standards Act and generates over 80 h of monthly overtime work.

Long work hours that continue for several months are called the “death by overwork level” because of the strong correlation with mental disorders and the onset of circulatory organ diseases, and it is a criterion for the recognition of labor accidents [14, 15]. This study’s results suggest that 68% of the respondents work in an environment exceeding the “death by overwork level.”

The Japanese Ministry of Health, Labour and Welfare decided to implement “Physician Labor Reform” in April 2024; the principle annual upper limit of overtime work for physicians shall be 960 h, 1860 h in specific cases, and various initiatives to achieve this goal are underway [16]. The plan promotes shifting tasks to other hospital staff and sets a continuous work time limit (28 h) with 9-hour intervals between work shifts and an upper limit for overtime hours [12].

For the 1860-hour cases, the average weekly in-hospital time was estimated to be < 80 h. This study’s results suggested that 27% of respondents worked hours that exceeded this criterion. Because such long working hours will become illegal in April 2024, measures must be taken to shorten working hours as soon as possible. However, the background factors for such super-long work hours have not been identified.

Long work hours of ≥ 80 in-hospital hours per week exhibited a significant correlation with males and no children, whereas no correlation with marital status was observed. The presence of children may require shorter work hours due to the time spent on childcare. Another possibility is that long work hours are not inhibited by the time invested in married life. Regarding sex, a report on American female surgery residents suggests that they have a higher proportion of long work hours and higher rates of experiencing burnout [16]. However, in this study, males had a stronger correlation with > 80 h of work per week. This is affected by the higher proportion of Japanese males in specialized areas that have long work hours, such as surgery and neurosurgery [17]. In this study, when controlling for specialty on multivariate analyses, male gender was still associated with long work hours.

Regarding age, the odds ratio was higher at ≥ 35–<40 years of age; 5% of participants belonged to this age group. These individuals may have become physicians later, or become residents again after changing their specialization, but the correlation with excessively long work hours is unclear. Further studies, such as additional surveys and interviews, will be necessary to explain why these physicians often work excessively long hours and identify other background factors.

Differences in long work hours were based on specialization areas. For example, surgeons often have long work hours. The Japan Surgical Society has stated that promoting initiatives such as drastic task shifting and consolidation of surgeries are necessary to shorten overtime hours. However, deeper discussions on self-improvement are necessary to ensure that reducing work hours does not impair surgeons’ acquisition of knowledge and skills [18]. In several countries, discussions are occurring regarding restricting work hours and whether policies that promote uniform work hour restrictions, regardless of specialization, are not appropriate [19].

In Japan, each physician can select their specialization both in medical school and after graduation [20]. Therefore, it is difficult to eliminate the uneven distribution of physician specialties. Physicians may also transfer from their selected, busy specialty to a less busy one [21]. According to the Japanese Ministry of Health, Labour and Welfare, the lowest proportion of physicians is in surgery and neurosurgery, where this study’s results showed significantly longer working hours. In contrast, there is a surplus in ophthalmology and dermatology, where long work hours are significantly scarce.

No significant correlation was observed between ≥ 80 in-hospital hours per week and the residents’ income. As stated previously, the annual pay of residents is low, and some physicians are even unpaid [11]. Therefore, because physicians’ long work hours are not necessarily compensated by greater pay, annual income may not be regarded as a background factor.

The regulations for residents’ work hours in the “Labor Reforms for Physicians” being advanced by the Japanese Ministry of Health, Labour and Welfare are based on those implemented by the US ACGME [22]. These regulations comprise 80 working hours per week, a continuous working time limit of 16 h, and a 10-hour interval between working hours. Contrastingly, the Japanese reforms comprise an upper limit of 1860 h on annual overtime, a 28-hour limit on continuous working time, and a 9-hour interval between working hours.

In the US, various evidence-based reviews have been performed on the ACGME regulations on residents’ work hours. Comparative reports regarding residents in surgery and internal medicine, using an intervention group without the continuous work time and shift interval restrictions and a control group using the ACGME regulated restrictions, indicated no significant difference in patient outcomes or residents’ degrees of satisfaction [23, 24]. The two studies cited were large. However, they had significant limitations, such as power to detect changes in mortality and outcomes metrics indirectly linked to study populations of interest, and the findings generally ran counter to the broader literature on the effects of efforts to reduce work hours on patient safety.

Another study in 2005 shows extended-duration work shifts (over 24 h), which were sanctioned by ACGME, pose safety hazards for interns [25]. In 2011, ACGME instituted a continuous working time limit of 16 h for residents. A recent study shows the 2011 ACGME work-hour limit was associated with meaningful improvements in physician safety and health [26]. Implementing such studies and examining evidence-based systemic reviews from the perspectives of patient safety, physician health, physician training, and so forth, is necessary in Japan.

Japanese resident physicians tend to have a low annual income. In this study, 52% of respondents had an annual income of less than 48,000 dollars (not including part-time work), and 70% were working part-time. Part-time workers are assumed to need to engage in part-time work at medical institutions other than their regular employer to be financially stable or to repay loans. Additionally, some doctors work at university hospitals as “unpaid doctors” [11]. Therefore, the need to improve residents’ remuneration can also be regarded as an issue.

If part-time work is treated as one of the causes of residents’ overwork, then the reduction of part-time work would be one method of decreasing residents’ work hours by promoting labor reforms for physicians. However, this may further reduce residents’ incomes. Therefore, there should also be proposals for pay supplementation using the national budget.

Additionally, medical institutions that have maintained their treatment systems so far through the part-time employment of residents must remember that treatment systems may become unsustainable with the termination of part-time work. Moreover, consideration should be given at the policy level so that local medical care is not affected, particularly in regions with a shortage of doctors.

This study has several limitations. First is the possibility of selection bias due to the individuals’ voluntary participation. Despite this limitation, effective responses were obtained from 4306 participants at 416 hospitals out of the 924 core hospitals nationwide accredited by the Japanese Medical Specialty Board (response rate = 49%). The response rate is related to the % of hospitals from which the responses were received. The number of residents and the location and specialty are unknown. Moreover, both the response rate and the number of respondents ensured a sample that was representative of the population.

Second, the survey used self-report responses, which may have introduced various information biases. For example, misreporting was possible because the in-hospital hours per week were not a detailed time study filled out by another person. Therefore, it couldn’t be confirmed whether the in-hospital hours were all work hours. Additionally, because participation in this survey was voluntary, detailed definitions of the specialist jargon used were not provided in order to increase the response rate.

Third, a statistical correlation was observed between long work hours and background factors. However, the causal relationship was unclear. It is possible that probable confounding factors were not measured in this study. Additionally, the effects of the COVID-19 pandemic were not examined. As shown in previous studies, the COVID-19 pandemic has greatly affected how physicians work [27], and it would be useful to conduct the same survey after the COVID-19 pandemic and compare the results of the studies. Furthermore, because there have been no previous similar nationwide studies on Japanese residents, it is difficult to compare the results of this study with the situation of residents before the COVID-19 pandemic.

The harsh working conditions of resident physicians in Japan and the background factors of longer work hours have been elucidated in tandem with discussions of solutions. To ensure the health of resident physicians, the quality of training, and the safety of patients, it is necessary to ascertain the status of resident physicians in greater detail, including vigorously promoting and verifying the effects of labor reforms for physicians.

## Conclusion

Our national survey shows that many residents work very long hours (more than 80 h/week), with some specialists working even longer hours, while others are required to work part-time jobs in order to be financially stable. Moreover, our results show a variety of background factors that correlate to working very long hours, such as being male, being between 30 and 40 years old, having no children, being in the surgical or neurosurgical specialty, and the number of physicians in the hospital. By understanding the factors that increase the likelihood of residents working very long hours, targeted changes can be made to address the specific concerns. Reducing working hours to a reasonable level can improve the resident physicians’ health and the quality of care they provide in their community.

## Data Availability

The datasets used and/or analyzed during the current study are available from the corresponding author on reasonable request.

## List of abbreviations

ACGME: Accreditation Council for Graduate Medical Education.
